# Safety of factor XI inhibitors compared to factor X inhibitors in atrial fibrillation: a systematic review and meta-analysis

**DOI:** 10.1007/s11239-025-03142-x

**Published:** 2025-07-03

**Authors:** Rafaella I. L. Markides, Sogol Koolaji, Joshua H. Leader, Mohamed Farag, Diana A. Gorog

**Affiliations:** 1 School of Clinical Medicine, University of Cambridge, Cambridge, UK; 2 Department of Cardiology, East and North Hertfordshire NHS Trust, Stevenage, Hertfordshire UK; 3 Faculty of Medicine, National Heart and Lung Institute, Imperial College, London, UK; 4 Cardiothoracic Department, Freeman Hospital, Newcastle-upon-Tyne, UK; 5 University of Hertfordshire, Hatfield, Hertfordshire UK; 6 School of Cardiovascular and Metabolic Medicine & Sciences, Kings College London, London, UK; 7 National Heart and Lung Institute Imperial College, Dovehouse Street, London, SW3 6LY UK

## Abstract

Atrial fibrillation (AF) increases the risk of ischemic stroke (IS) and systemic embolism, necessitating thromboprophylaxis with direct oral anticoagulants (DOAC), which increase bleeding. Drugs that inhibit factor XI (FXI) have been developed to provide thromboprophylaxis with lower bleeding risk. We performed a systematic review and meta-analysis of randomised controlled trials comparing FXI inhibitors versus DOAC in patients with AF, reporting primary outcomes of International Society of Thrombosis and Haemostasis (ISTH) major bleeding or clinically relevant non-major bleeding (CRNMB), and exploratory outcomes of ischaemic stroke (IS), intracranial hemorrhage (ICH) and death. Three trials were identified. The PACIFIC-AF (Phase 2) and OCEANIC-AF (Phase 3) trials compared asundexian, an oral, small-molecule FXIa inhibitor, with apixaban. AZALEA-TIMI 71 (Phase 2) compared abelacimab, a subcutaneous monoclonal antibody against FXI/FXIa, with rivaroxaban. FXI inhibitors reduced the composite of major bleeding or CRNMB (pooled-OR 0.39, 95%CI 0.30–0.50, *p* = 0.0005), major bleeding (OR 0.30, 95%CI 0.22–0.41, *p* = 0.004) and CRNMB (pooled-OR 0.44, 95% CI 0.36–0.55, *p* = 0.0004) compared to DOAC. Effects were consistent across sex, clinical risk factors and concomitant antiplatelet therapy. Exploratory analyses showed FXI inhibitor use was associated with greater risk of IS (OR 3.37, 95%CI 2.18–5.19, *p* = 0.001), similar rate of ICH and lower all-cause mortality (OR 0.82, 95%CI 0.71–0.94, *p* = 0.02) than DOAC. Compared to DOAC, FXI inhibitors significantly reduced major bleeding or CRNMB. Exploratory analyses indicate similar risk of ICH, but possible increased IS risk with FXI inhibitors compared to DOAC. Results from ongoing trials will help determine the relative usefulness of FXI inhibitors in patients with AF.

**Supplementary Information:**

The online version contains supplementary material available at 10.1007/s11239-025-03142-x.

## Graphical Abstract

**Supplementary Information:**

The online version contains supplementary material available at 10.1007/s11239-025-03142-x.

## Graphical abstract

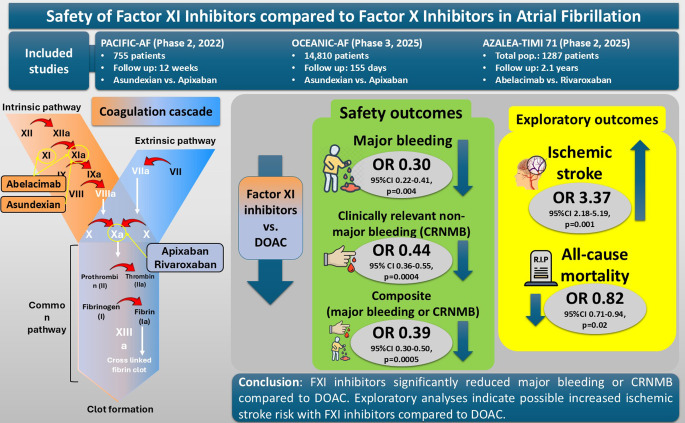

## Introduction

Atrial fibrillation (AF) increases the risk of ischemic stroke and systemic embolism five-fold, necessitating treatment with anticoagulation, as thromboprophylaxis against thrombotic events, in the majority of patients. Guidelines recommend the use of direct-acting oral anticoagulants (DOACs) over vitamin K antagonists, owing to their greater safety and efficacy [1]. However, while safer than vitamin K antagonists, DOACs nevertheless carry a significant risk of major bleeding, with annual rates reported at between in 2–4% in randomised controlled trials [2–4] and 2–5% in real world registries [5, 6]. Additionally, there are a significant number of individuals who have contraindications to anticoagulation because of an excessively high bleeding risk or who are reluctant to take OAC due to the fear of bleeding complications. Such individuals are exposed to a very high risk of stroke and systemic embolism. There is, therefore, an unmet need for an anticoagulant that provides thromboprophylaxis in patients with AF but with a lower risk of bleeding than that afforded by current DOACs.

Interest in targeting factor XI (FXI) as an anticoagulant strategy stems from observational data in humans showing that individuals with congenital FXI deficiency (haemophilia C) have only mild increase in bleeding in response to trauma and do not exhibit spontaneous bleeding events or increased risk of intracranial hemorrhage, but at the same time have a low incidence of stroke and venous thromboembolism (VTE) [7, 8]. This finding, that FXI depletion may protect from VTE in the absence of extensive bleeding, is supported by laboratory experiments. Deletion of FXI in mice reduced arterial thrombus formation in a carotid artery model without increasing bleeding [9], and in other animal models (rabbit, monkey, baboon) reduced FXI levels attenuated thrombosis without causing bleeding [9–12]. 

Thrombosis in vivo is either triggered by low concentrations of tissue factor (TF) exposed at the sites of endothelial disruption, or by contact with the artificial surfaces of medical devices that bind FXII. This leads to the production of thrombin, which in turn, through a positive feedback mechanism, further activates FXI, amplifying the formation of thrombin and fibrin, leading to thrombus growth. Events leading to hemostasis are initiated through the TF pathway, leading to the downstream activation of FX, resulting in thrombin generation and ultimately the formation of a fibrin clot. However, the absence of a positive feedback loop in the hemostatic pathway means that amount of fibrin produced is limited and sufficient to achieve hemostasis, without culminating in thrombosis. This differential role for FXI, playing an important part in thrombosis but a much smaller role in hemostasis, led to the concept that inhibition of FXI activity may be an attractive antithrombotic therapeutic strategy [13, 14]. Pharmacotherapies currently being evaluated in clinical trials that target FXI do so either by reducing FXI biosynthesis or by directly inhibiting FXI/FXIa [15].

Following earlier randomised clinical trials evaluating the safety and efficacy of FXI inhibition in patients at risk of VTE, more recent trials have investigated these agents in other settings, namely in patients with non-valvular AF, acute coronary syndrome and stroke. Whilst there are ongoing phase 3 trials in patients with AF to assess the effectiveness of FXI inhibition in preventing thromboembolic complications, it was our aim to assess the existing available data from clinical trials regarding the comparative safety of FXI inhibitors in comparison to DOACs, in individuals with AF.

## Methods

This review was conducted in accordance with the guidelines set by Preferred Reporting Items for Systemic Reviews and Meta-analyses (PRISMA). The study was registered on the PROSPERO database (CRD 420250654263).

### Search strategy and data extraction

Digital databases (PubMed and Cochrane Library) were searched from inception through to 3 March 2025, using various combinations of medical subject headings (MeSH) (Supplementary List 1). The subsets were combined in various combinations, with the search restricted to full-length articles published in English in peer-reviewed journals. Abstracts were screened and potentially relevant articles underwent full-text review.

Two reviewers (RM and SK) independently reviewed all titles, or titles and abstracts to identify articles that met the study inclusion criteria, with backward snowballing to retrieve studies that were missed on the initial database search. Selected studies were compared, and disagreement resolved by discussion and consensus. Data extraction was performed independently and in duplicate by the study investigators. Articles selected for the final review were checked to avoid inclusion of duplicate data. Data were collected from each study on baseline characteristics, concomitant antiplatelet therapy, and efficacy and safety clinical outcomes.

### Inclusion and exclusion criteria

We included randomised controlled trials only, restricted to human studies only, published in English and reporting bleeding events.

### Study endpoints

The primary efficacy endpoint was the safety profile of FXI inhibitors in patients with AF, compared to DOACs, including outcomes of major bleeding, clinically relevant non-major (CRNM) bleeding as classified by the International Society of Thrombosis and Haemostasis (ISTH). Secondary endpoints included ischemic stroke, stroke or systemic embolism, intracranial hemorrhage and death.

### Statistical analysis

Outcomes were pooled using crude number of events retrieved from each study and compared using a fixed-effect or random-effect model according to the heterogeneity among the included studies. Treatment effect was reported as odds ratio (OR) and 95% confidence interval (CI). Pooled ORs with 95% CI were estimated for binary variables using a random-effects Mantel Hanzel model with the method of DerSimonian and Laird [16]. Hartung-Knapp-Sidik-Jonkman (HKSJ) method was applied for calculating 95%CI. Heterogeneity between individual studies was explored by χ2 statistic and characterized with I [2] statistic. A p-value < 0.05 was considered statistically significant.

Included studies were assessed using the Cochrane risk-of-bias tool by two authors. As recommended by the Cochrane handbook of systematic reviews and meta-analysis, we performed the quality assessment of each study using the Cochrane risk of bias tool. Publication bias such as funnel plot, or Egger’s test is not applicable due to low number of studies. In addition to a comprehensive analysis of all strategies, we performed a meta regression to analyse trials separately based on the type of antiplatelet strategy, patient sex, chronic kidney disease, coronary artery disease, hypertension and heart failure.

Primary analyses were performed using RevMan Version 5.3.5 software (The Nordic Cochrane Centre, The Cochrane Collaboration, 2014), and “metafor” and “meta” packages in R version 4.3.2 software for meta-regression, and leave-one-out analysis. The leave-one-out analysis was performed to assess sensitivity.

## Results

### Baseline characteristics

**Fig. 1:**
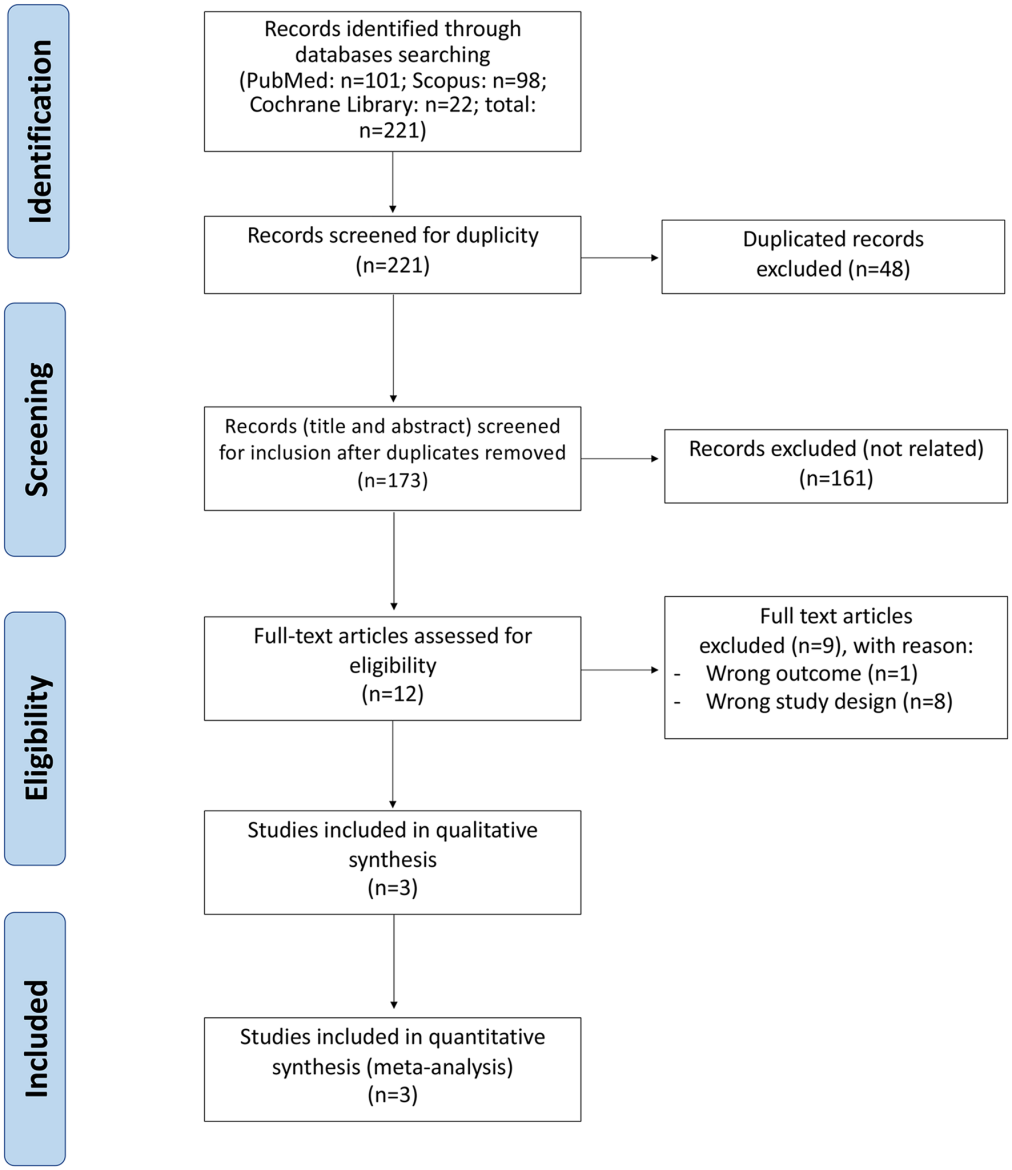
PRISMA diagram

A total of 221studies were identified, leaving 173 after removal of duplicates. A further 161were excluded after review of the title and/or abstract (Fig. 1). Three trials met our inclusion and exclusion criteria, namely PACIFIC-AF [17], OCEANIC-AF [18] and AZALEA-TIMI 71 [19]. All three were international, multicentre RCTs. The first two were designed as double-blinded but third one was designed as partially blinded study (open-label with respect to drug allocation, but patients and investigators were blinded to the investigational drug dose) (Table 1).

**Table 1:** Study characteristics and baseline data

Name of trial	PACIFIC-AF, 2022			OCEANIC-AF, 2025		AZALEA-TIMI 71, 2025		
Study drug & dose	Asundexian 20 mg od	Asundexian 50 mg od	Apixaban 5 mg bd	Asundexian 50 mg od	Apixaban 5 mg bd	Adelacimab 90 mg o/m	Adelacimab 150 mg o/m	Rivaroxaban 20 mg od
**Study characteristics**								
Number of patients	251	254	250	7415	7395	427	430	430
Trial design	Multinational, randomised, double-blind, double-dummy, dose-finding phase 2 trial			Multinational, randomised, double-blind, double-dummy, parallel-group, active comparator-controlled phase 3 trial		Multinational, randomised, partially blind, parallel-group, active-controlled phase 2b trial		
Duration of treatment	12 weeks			Stopped prematurely		Stopped prematurely Total planned trial duration 27 months		
Median follow up (IQR)	12 weeks (overall 671 patient completed treatment phase)			Median: 155 days		At the time of recommended termination: 1.8 years (1.7–1.9); At trial completion: 2.1 (2.0-2.3)		
Primary endpoint	Composite ISTH major or clinically relevant non-major bleeding			ISTH major bleeding		Composite ISTH major or clinically relevant non-major bleeding		
**Drug characteristics**								
Type of agent	Small molecule	Small molecule	Small molecule	Small molecule	Small molecule	Fully human monoclonal antibody	Fully human monoclonal antibody	Small molecule
Mode of action	Factor XIa inhibitor	Factor XIa inhibitor	Factor Xa inhibitor	Factor XIa inhibitor	Factor Xa inhibitor	Factor XI inhibitor	Factor XI inhibitor	Factor Xa inhibitor
Route of administration	Oral	Oral	Oral	Oral	Oral	Subcutaneous	Subcutaneous	Oral
**Patient characteristics**								
Age mean (SD) Median (IQR)	73.6 (8.0)	73.1 (8.5)	74.3 (8.3)	73.9 (7.7)	73.9 (7.7)	*75 (69–79)	*74 (69–78)	*74 (69–79)
Female, n (%)	103 (41)	97(38)	109 (44)	2656 (35.8)	2558 (34.6)	195 (45.7)	193 (44.9)	184 (42.8)
CHA_2_DS_2_-VASc score, Mean (SD) *Median (IQR)	3.9 (1.4)	3.8 (1.3)	4.1 (1.4)	4.3 (1.3)	4.3 (1.3)	*5.0 (4.0–5.0)	*5.0 (4.0–5.0)	*5.0 (4.0–6.0)
Single antiplatelet therapy, n (%)	35 (14)^a^	33 (13)^a^	39 (16)^a^	742 (10.0)^b^	743 (10.0)^b^	98 (23.0)	99 (23.0)	100 (23.3)
HAS-BLED score, median (IQR)	--	--	--	--	--	2.0 (2.0–3.0)	2.0 (2.0–3.0)	3.0 (2.0–3.0)
**Pattern of atrial fibrillation**								
First detected or paroxysmal, n/total n (%)	122/196 (62.2)^c^	115/188 (61.2)^c^	117/182 (64.3)^c^	2878/7474 (38.8)	2775/7392 (37.5)	224/426 (52.6)	220/424 (51.9)	225/428 (52.6)
Persistent or long-standing persistent, n/total n (%)	74/196 (37.8)	73/188 (38.2)	65/182 (35.7)	2209/7414 (29.6)	2233/7392 (30.2)	87/426 (20.4)	84/424 (19.8)	97/428 (22.7)
Permanent, n/total n (%)	--	--	--	2327/7415 (31.4)	2384/7395 (32.2)	115/426 (27.0)	20/424 (28.3)	106/428 (24.8)
**Comorbidities**								
Hypertension, n (%)	226/251 (90)	227/254 (89)	220/250 (88)	6558/7415 (88.4)	6565/7395 (88.8)	410/427 (96.0)	417/430 (97.0)	418/430 (97.2)
Heart failure, n (%)	108/251 (43)	107/254 (42)	117/250 (47)	3456/7415 (46.6)	3473/7395 (47.0)	192/427 (45.0)	182/430 (42.3)	206/430 (47.9)
Coronary artery disease, n (%)	76/251 (30)	71/254 (28)	85/250 (34)	2496/7415 (33.7)	2452/7395 (33.2)	218/427 (51.1)	199/430 (46.3)	205/430 (47.7)
Diabetes, n (%)	83/251 (33)	74/254 (29)	87/250 (35)	2722/7415 (36.7)	2748/7395 (37.2)	223/427 (52.2)	231/430 (53.7)	245/430 (57.0)
Previous stroke/TIA, n (%)	22/251 (9)	18/254 (7)	25/250 (10)	1389 /7415 (18.7)	1305/7395 (17.6)	95 (22.3) [57/427 (13.3) stroke; 38/426 (8.9) TIA]	84 (19.5) [59/430 (13.7) Stroke; 25/429 (5.8) TIA]	107/429 (24.9)
Chronic kidney disease, n (%)	55/251 (22)^d^	84/254 (33)^d^	77/250 (31)^d^	1399/7415 (18.9)^e^	1357/7395 (18.4)^e^	86/425 (20.2)^f^	90/430 (20.9)^f^	88/429 (20.5)^f^

a: Aspirin ≤ 100 mg/day

b: For > 6 months

c: Only paroxysmal AF

d: Excluded eGFR < 30

e: Excluded eGFR < 25

f: Excluded eGFR < 15

The PACIFIC-AF (Phase 2) and OCEANIC-AF (Phase 3) trials compared asundexian [17, 18], an oral, small-molecule anticoagulant that inhibits activated factor XI (FXIa) with twice daily apixaban. AZALEA-TIMI 71^19^ was a Phase 2 trial assessing the abelacimab, a fully human, monoclonal antibody designed bind to the catalytic domain of factor XI and prevent its activation to FXIa, given subcutaneously once a month, compared with once daily rivaroxaban (Table 1). The demographics of participants, including age, sex, type of AF, and clinical risk factors including hypertension, diabetes mellitus, prior stroke or transient ischemic attack, chronic kidney disease, heart failure, as well as CHA_2_DS_2_VASC and HAS BLED scores, are reported in Table 1.

The primary safety endpoint in the two phase 2 trials was the composite of ISTH major bleeding or CRNM bleeding, while the primary safety endpoint in OCEANIC-AF was ISTH major bleeding (Table 2). Other reported exploratory endpoints are summarized in Table 3.

**Table 2:** Primary and secondary safety endpoints

	PACIFIC-AF, 2022			OCEANIC-AF, 2025		AZALEA-TIMI 71, 2025		
Study drug & dose	Asundexian 20 mg od	Asundexian 50 mg od	Apixaban 5 mg bd	Asundexian 50 mg od	Apixaban 5 mg bd	Adelacimab 90 mg o/m	Adelacimab 150 mg o/m	Rivaroxaban 20 mg od
Primary safety endpoints:								
ISTH major bleeding, n (%)	0/251 (0)	0/254 (0)	0/250 (0)	17/7373 (0.2)	53/7364 (0.7)	8/425 (1.9)	10/427 (2.3)	31/428 (7.2)
ISTH clinically relevant non-major bleeding, n (%)	3/251 (1)	1/254 (< 1)	6/250 (2)	67/7373 (0.9)	140/7364 (1.9)	13/425 (3.1)	16/427 (3.7)	35/428 (8.2)
Composite ISTH major or clinically relevant non-major bleeding, n (%)	3/251 (1)	1/254 (< 1)	6/250 (2)	83/7373 (1.1)	188/7364 (2.6)	21/425 (4.9)	26/427 (6.1)	66/428 (15.4)
Secondary safety endpoints:								
Any adverse event, n (%)	118/249 (47)	120/254 (47)	122/250 (49)	2573/7373 (34.9)	2569/7364 (34.9)	358/425 (83.8)	351/427 (82.6)	348/428 (81.3)
Any study drug-related adverse event, n (%)	29/249 (12)	26/254 (10)	37/250 (15)	385/7373 (5.2)	502/7364 (6.8)	--	--	--
Any adverse event leading to discontinuation of trial drug, n (%)	15/249 (6)	16/254 (6)	13/250 (5)	147/7373 (2.0)	118/7364 (1.6)	32/425 (7.5)	29/427 (6.8)	29/428 (6.8)
Any serious adverse event, n (%)	22/249 (9)	20/254 (8)	13/250 (5)	582/7373 (7.9)	599/7364 (8.1)	158/425 (37.2)	157/427 (36.8)	167/428 (39.0)
Any study drug-related serious adverse event, n (%)	4/249 (2)	0/254 (0)	0/250 (0)	27/7373 (0.4)	47/7364 (0.6)	--	--	--
Any serious adverse event leading to discontinuation of trial drug, n (%)	4/249 (2)	4/254 (2)	4/250 (2)	38/7373 (0.5)	35/7364 (0.5)	--	--	--
Adverse event with outcome of death, n (%)	1/249 (< 1)	3/254 (1)	2/250 (1)	29/7373 (0.4)	43/7364 (0.6)	--	--	--
All-cause mortality, n (%)	2/251 (< 1)	4/254 (2)	4/250 (2)	60/7415 (0.8); 73 (1.0)^a^	71/7395 (1.0); 85 (1.2)^a^	26/425 (6.1)	22/427 (5.2)	30/428 (7.0)
Gastrointestinal bleeding, n (%)	--	--	--	--	--	2/425 (0.5)	2/427 (0.5)	18/428 (4.2)
Intracranial haemorrhage, n (%)	--	--	--	*3/7373 (< 0.1)^b^	18/7364 (0.2)^b^	4/425 (0.9)	2/427 (0.5)	4/428 (0.9)
Injection site reaction, n (%)	--	--	--	--	--	7/425 (1.6)	12/427 (2.8)	N/A

a: In full safety population at the end of follow up (beyond the end of treatment)

b: Symptomatic intracranial haemorrhage

**Table 3:** Exploratory endpoints

	PACIFIC-AF, 2022			OCEANIC-AF, 2025		AZALEA-TIMI 71, 2025		
Study drug & dose	Asundexian 20 mg od	Asundexian 50 mg od	Apixaban 5 mg bd	Asundexian 50 mg od	Apixaban 5 mg bd	Adelacimab 90 mg o/m	Adelacimab 150 mg o/m	Rivaroxaban 20 mg od
Ischaemic stroke, n (%) Events/100 patient-yr (95% CI)	2/251(< 1)	1/254 (< 1)	0/250 (0)	85/7415 (1.1) *3.34 (2.67–4.08)	21/7395 (0.3) *0.82 (0.51–1.21)	10/425 (2.4) *1.24	10/427 (2.3) *1.21	5/428 (1.2) *0.59
Systemic embolism, n (%) Events/100 patient-yr (95% CI)	0/251 (0)	0/254 (0)	0/250 (0)	--	--	0/425 (0) *0	1/427 (0.2) *0.12	0/428 (0) *0
Stroke or systemic embolism, n (%) *Events/100 patient-yr (95% CI)	--	--	--	98/7415 (1.3) *3.85 (3.13–4.65)	26/7395 (0.4) *1.02 (0.66–1.44)	11/425 (2.6) *1.36	10/427 (2.3) *1.21	7/428 (1.6) *0.83
Death from cardiovascular cause, n (%) *Events/100 patient-yr (95% CI)	1/251 (< 1)	3/254 (1)	3/250 (1)	48/7415 (0.6) *1.87 (1.38–2.44)	44/7395 (0.6) *1.72 (1.25–2.26)	--	--	--
Myocardial infarction, n (%)	0/251 (0)	1/254 (< 1)	0/250 (0)	--	--	--	--	--

All included studies were assessed to have a low risk of bias across all domains using the Cochrane risk-of-bias tool, indicating high methodological quality (Supplementary Fig.0;1).

### Meta-analysis of bleeding outcomes

**Fig. 2:**
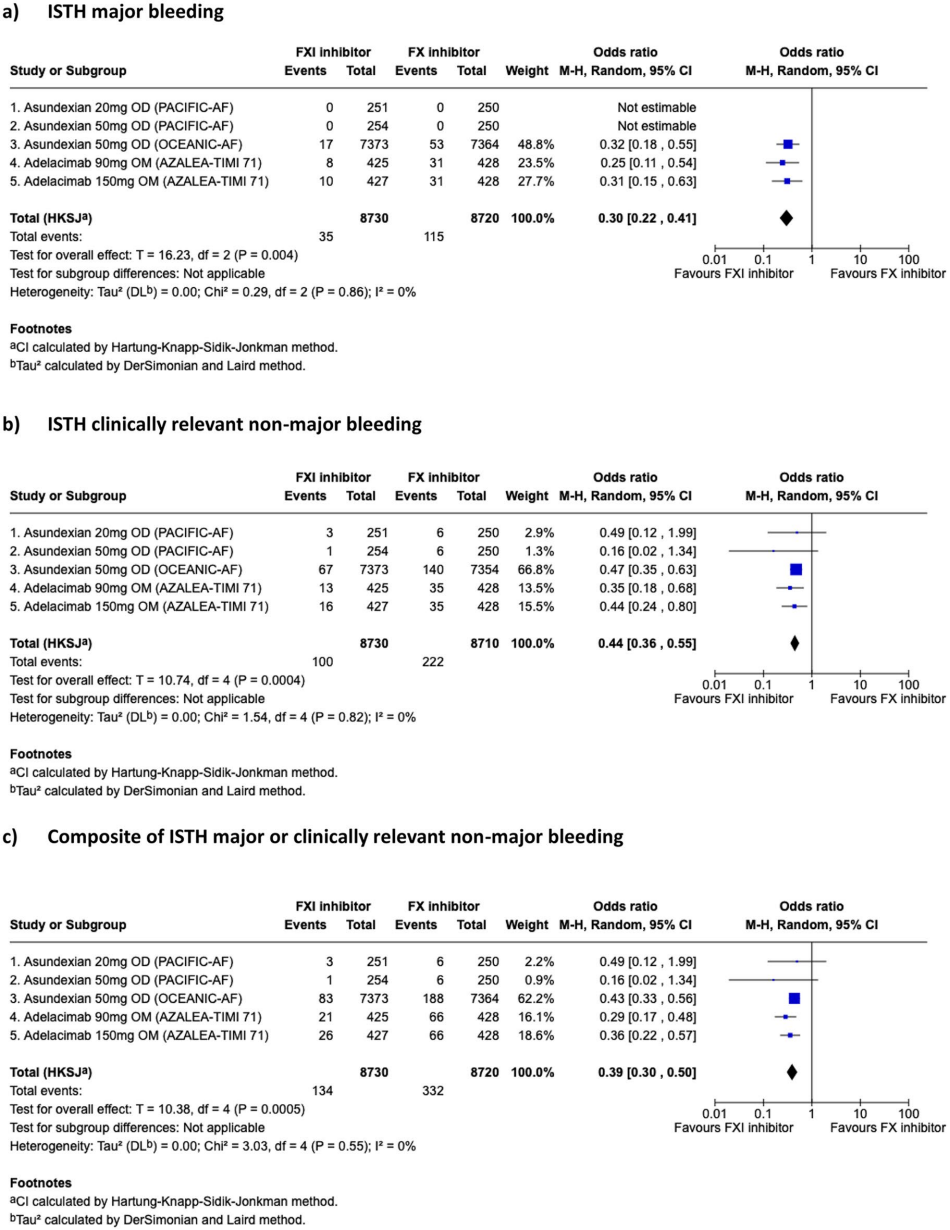
Primary endpoints of meta-analysis. ISTH clinically relevant non-major bleeding Composite of ISTH major or clinically relevant non-major bleeding

**Fig. 3:**
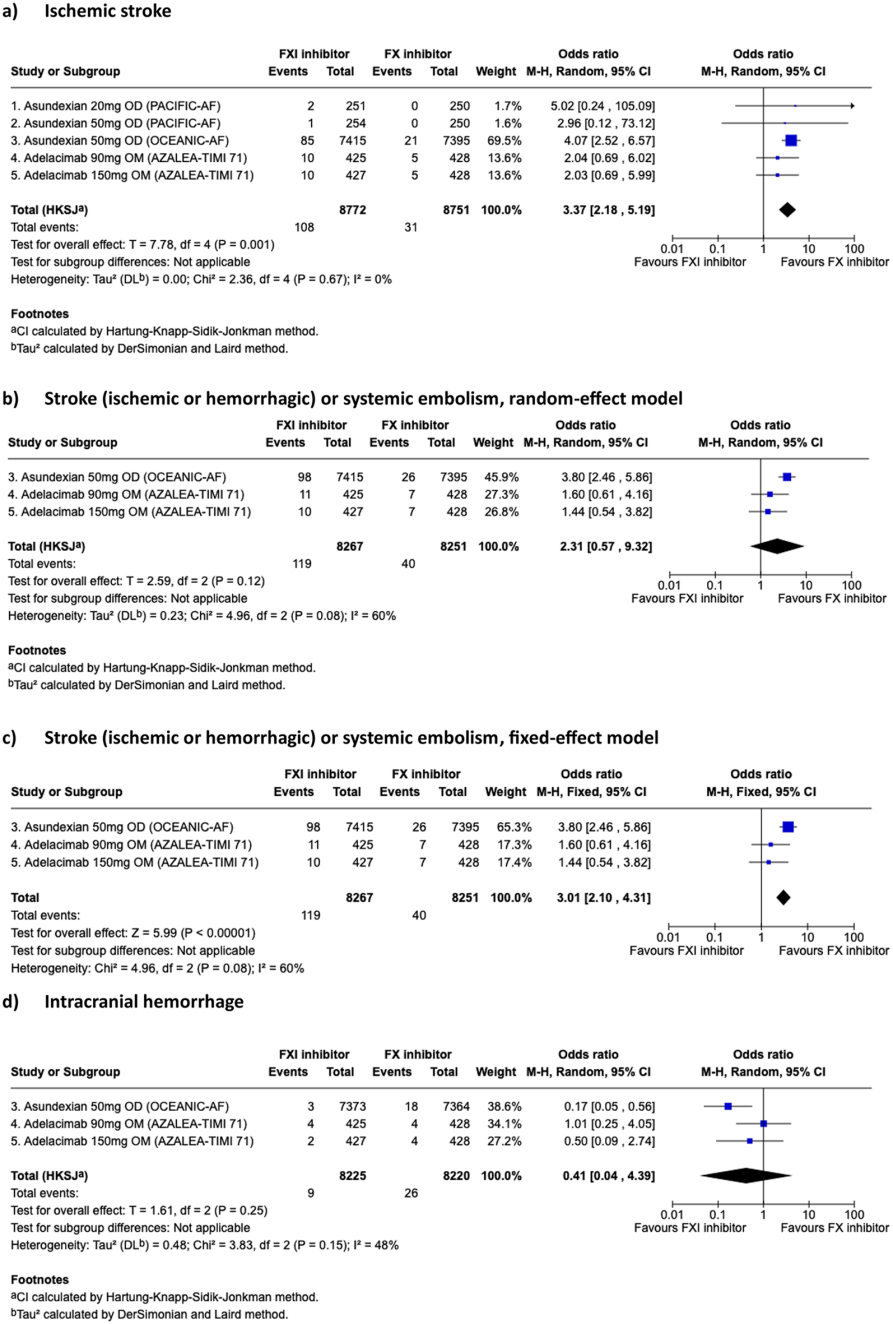
Exploratory outcomes meta-analysis

Across all doses, FXI inhibitor significantly reduced the composite of ISTH major bleeding or CRNM bleeding compared to DOAC (pooled-OR 0.39, 95%CI, 0.30–0.50, *p* = 0.0005) and CRNM bleeding to DOAC (pooled-OR 0.44, 95% CI 0.36–0.55, *p* = 0.0004) (Fig. 2a-c). Asundexian 50 mg or abelacimab at both doses reduced ISTH major bleeding compared to DOAC (OR 0.30, 95%CI 0.22–0.41, *p* = 0.004). There was no evidence of statistical heterogeneity between studies for all of these three outcomes (i^2^ = 0%). Numerically, the occurrence of ICH was lower with FXI inhibitor than with DOAC but this was not statistically significant (OR 0.41, 95%CI 0.04–4.39, *p* = 0.25) (Fig. 3d). There was moderate heterogeneity between the studies (i^2^ = 48%) although Cochrane’s Q test was not statistically significant (*p* = 0.15) (Fig. 3d).

### Meta analysis of thromboembolic outcomes and mortality

**Fig. 4:**
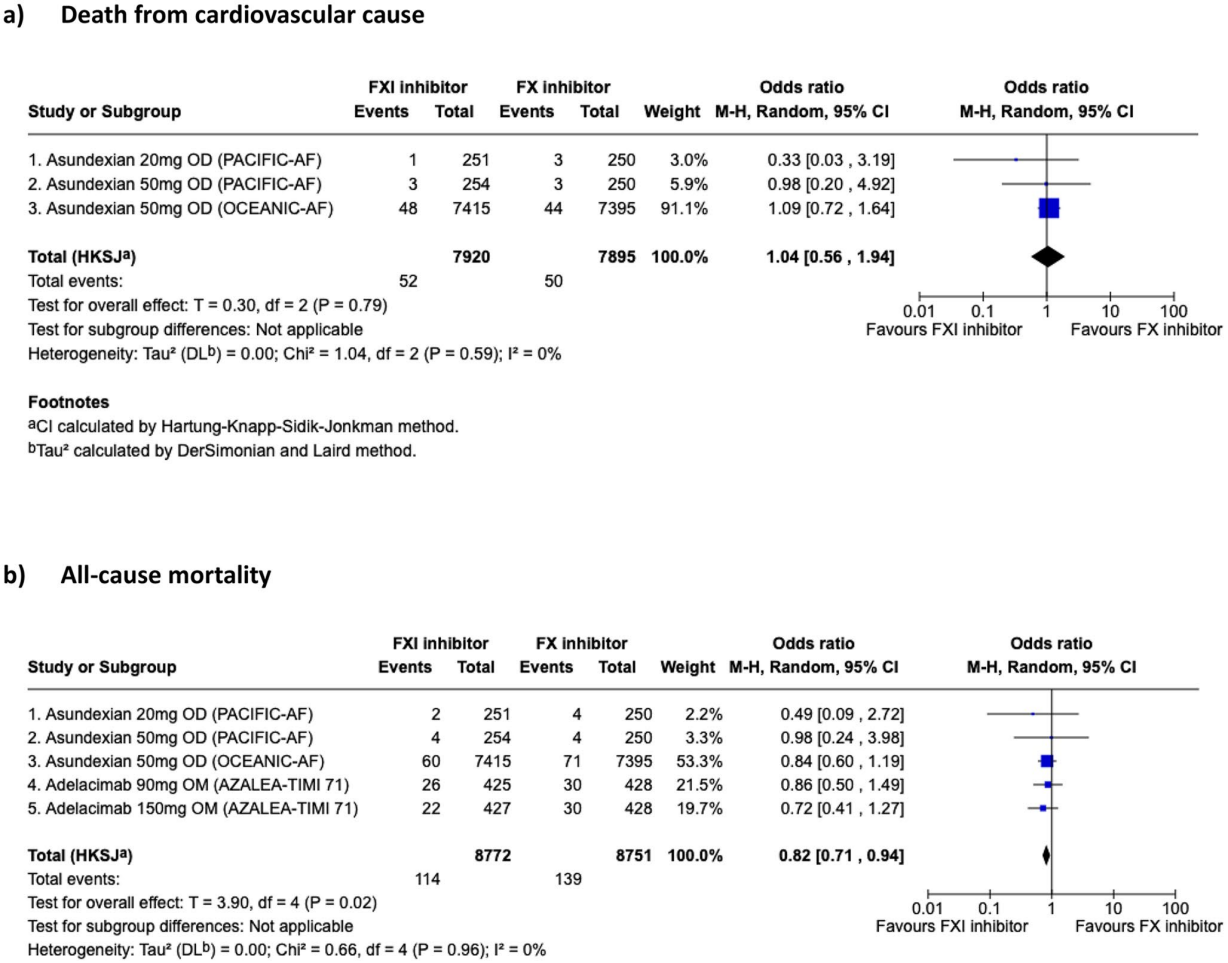
Exploratory outcomes of death. Death from cardiovascular cause All-cause mortality

Ischemic stroke occurred more often with FXI inhibitor versus DOAC (OR 3.37, 95%CI 2.18–5.19, *p* = 0.001) (Fig. 3a). No statistical heterogeneity was observed between studies (i^2^ = 0%, *p* = 0.67). The occurrence of the composite of stroke (ischemic and hemorrhagic) and systemic embolism was not statistically different between FXI inhibitor and DOAC in a random-effect model (OR 2.31, 95%CI 0.57–9.32) but with a heterogeneity of 60% (*p* = 0.08) (Fig. 3b). To investigate the model sensitivity and understanding heterogeneity’s impact, we also tested a fixed-effect model that showed a statistically significant greater OR of composite of stroke (ischemic and hemorrhagic) and systemic embolism in FXI inhibitor arm compared to DOAC (OR 3.01, 95%CI 2.10–4.31) (Fig. 3c). All-cause mortality was lower with FXI inhibition than DOAC (OR 0.82, 95%CI 0.71–0.94, *p* = 0.02) (Fig. 4).

### Sensitivity analysis

The sensitivity analysis was conducted by removing one study at a time for all outcomes where applicable (leave-one-out analysis) (Supplementary Fig. 2). While most outcomes showed minimal change upon study exclusion, the pooled OR for ischemic stroke showed mild reduction by omitting OCEANIC-AF study, though the direction and significance of the effect remained consistent (pooled-OR after omitting the study, 2.22, 95%CI, 1.08–4.55, *p* = 0.03) (Supplementary Fig. 2). Furthermore, leaving OCEANIC-AF out for stroke or systemic embolism resulted in a significant reduction in pooled OR from 3.01 (95%CI 2.10–4.31) to 1.52 (95%CI, 0.77–3.01, *p* = 0.23) (Supplementary Fig. 2).

### Meta regression analysis

Meta-regression showed no statistically significant difference in the effects based on sex, chronic kidney disease, coronary artery disease, diabetes, heart failure or those on single antiplatelet therapy with respect to major bleeding, CRNM bleeding, composite of major bleeding and CRNM bleeding or ischemic stroke (all *p* > 0.05). As presented in the meta regression graphs, as these clinical risk factors increase, the log OR ischemic stroke declines (negative slope) although this difference was not statistically significant (*p* > 0.05). In contrast, the meta-regression demonstrated a positive association though not statistically significant between heart failure and the log OR ischemic stroke (positive slope, *p* = 0.23) (Supplementary Fig. 3).

## Discussion

To our knowledge, this is the first systematic review and meta-analysis of randomised controlled trials comparing FXI inhibition versus DOAC for thromboprophylaxis in patients with AF. FXI inhibitors were associated with a significant reduction in major bleeding, CRNM bleeding, and the composite of major and CRNM bleeding events, compared to DOACs. This result is particularly significant because it includes results from phase II studies, which naturally included subtherapeutic dosages of FXI inhibitors. The magnitude of relative risk reduction by FXI inhibition compared to DOAC was 70% for major bleeding events and 56% for CRNM bleeding. The results are consistent among the studies included in the meta-analysis, with absence of significant heterogeneity. Absolute rates of bleeding and major adverse cardiovascular events observed in this meta-analysis for DOACs are roughly in line with landmark clinical trials of thromboprophylaxis in patients with AF [5]. 

Since two of the 3 trials assessed here were not powered to assess ischemic endpoints, our secondary analyses have to be considered exploratory. Use of FXI inhibitor was associated with a 3.37-fold increased risk of ischemic stroke, but similar incidence of overall (ischemic and hemorrhagic) stroke and systemic embolism with no heterogeneity observed between studies. This raises concerns about the possible effectiveness of these drugs for thromboprophylaxis, compared to DOAC. However, notably, the Phase 2 trials, PACIFIC-AF and AZALEA-TIMI 71 were powered to assess bleeding (safety) and not ischemic events (efficacy for thromboprophylaxis) [17, 19]. Although using the fixed-effect model, the risk of stroke or systemic embolism was significantly greater with FXI inhibitor versus DOAC, excluding the OCEANIC-AF trial rendered the increased risk non-significant. The substantial heterogeneity observed across studies further highlights the discrepancy and the considerable influence of the OCEANIC-AF trial on the overall results. The reason for the increased risk of ischemic stroke seen with FXIa inhibition compared to DOAC is unclear, but some possible mechanisms are worthy of discussion. Firstly, in dose-finding studies, the effectiveness of asundexian was gauged by the degree of FXI inhibition. However, the extent of FXI inhibition may not directly reflect efficacy of the drug on thrombin generation or even on fibrin generation. A differential impact of apixaban and asundexian on thrombin generation is supported by pharmacodynamic studies in vitro [20]. Furthermore, pharmacokinetic studies showed that the inhibition constant (Ki) of asundexian for FXIa is 1.0 nM [20], whereas the Ki for apixaban against FXa is 0.08 nM [21, 22]. This may be pertinent for dosing. While the half-lives of milvexian and asundexian are roughly similar, the OCEANIC-AF trial with asundexian was terminated prematurely for non-inferiority while the phase 3 trial with milvexian, LIBREXIA-AF has finished recruiting and is now in follow-up(x), without premature closure due to efficacy concerns. However, it is noteworthy that the selected dose of milvexian in LIBREXIA-AF (ClinicalTrials.gov ID No. NCT05757869) at 100 mg twice daily is 4-fold higher than the dose of asundexian studied in OCEANIC-AF (50 mg daily). Furthermore, milvexian has been reported to have greater potency in the FXIa enzyme inhibition assay in vitro compared to that reported for asundexian [23, 24]. Lastly, perhaps the setting of AF may not be the right thrombotic target for FXI/FXIa inhibition. Since FXI inhibitors target the intrinsic pathway of coagulation, it is not surprising that early studies in vitro showed that asundexian was less effective at inhibiting thrombin generation in the presence of high concentrations of tissue factor (TF) compared to that when low amounts of TF were used as trigger [24]. It is possible that in patients with AF, the contribution of the extrinsic (TF) pathway of coagulation has been underestimated and TF may play a more significant role than previously thought.

All-cause mortality was lower with FXI inhibition than DOAC. The mechanism behind this is unclear, although likely to at least in part relate to the lower risk of major bleeding with FXI inhibition. Additionally, the lower rate of minor/nuisance bleeding reported with FXI inhibition could have resulted in better treatment compliance in patients assigned to FXI inhibitor than those taking DOAC, leading to lower risk of venous thromboembolism.

Finally, possible off-target beneficial effects of FXI inhibition have to be considered. FXI activation promotes a proinflammatory phenotype and FXI inhibition in non-human primates was shown to reduce C-reactive protein, platelet reactivity and endothelial cell activation [25]. Additionally, in vitro studies have shown that FXI activation increases vascular permeability, a recognized feature of inflammation, and inhibition of FXI activation was shown to preserve endothelial barrier function [26]. 

Meta-regression analyses revealed no statistically significant differences in bleeding outcomes, ischemic events, or mortality between FXI inhibitor and DOAC when stratified by sex, cardiovascular risk factors for ischaemic events, or concomitant use of antiplatelet therapy.

The relevance of these findings is that there is potentially a new anticoagulant strategy to reduce major bleeding compared to standard-of-care (FXa inhibitors), and whilst our study specifically analyzed patients with an indication for anticoagulation for thromboprophylaxis of AF, other patients with indications for anticoagulation may also benefit from such a strategy to reduce major bleeding. This includes patients with as venous thromboembolism, particularly patients with cancer, as well as for secondary prevention of stroke and in patients with acute coronary syndrome [27]. Such a strategy to reduce bleeding may be particularly relevant for patients with AF who have had a prior bleed on a DOAC, or are at increased risk of bleeding [28]. The risk of OAC-related bleeding increases with age and includes those with uncontrolled hypertension, solid tumors or hematological malignancies, liver or renal dysfunction, prior stroke or cerebral small vessel disease or amyloid angiopathy, those with anemia, thrombocytopenia, those the excessive alcohol intake or those who require concomitant antiplatelet medications. Additionally, there is undeniably a cohort who are undertreated with DOAC because of concerns around frailty and falls-risk, in whom the lower risk of bleeding afforded by FXIa inhibitor compared to that with DOACs, may favor the use of thromboprophylaxis. The exploratory analyses indicating similar risk of intracranial hemorrhage with FXI/FXIa inhibitors and DOACs, if borne out in the ongoing Phase 3 studies, would imply that use of FXIa inhibitors would not be preferred over DOACs in those with prior stroke, cerebral small vessel disease or amyloid angiopathy. Furthermore, DOACs would continue to be preferrable over FXI/FXIa inhibitors for those at high ischemic risk, and/or those at low risk of bleeding risk, but FXIa inhibitors may be preferred for those at lower ischemic and higher bleeding risks.

An ongoing large, double-blind, randomized Phase 3 trial (LIBREXIA-AF) is evaluating another small molecule oral FXIa inhibitor, milvexian, compared to asundexian, to reduce the risk of the composite stroke and non-central nervous system systemic embolism in ~ 20,000 patients with AF (ClinicalTrials.gov ID No. NCT05757869). The trial has finished recruiting and is in follow up. Importantly, compared to OCEANIC-AF, where asundexian was given as a once-a-day 50 mg dose, in LIBREXIA-AF, milvexian is given at a dose of 100 mg twice daily, in effect fourfold higher dose per 24 h period than for asundexian in OCEANIC-AF. This will help clarify the effectiveness and safety of higher dose oral FXIa inhibition compared to DOAC. Furthermore, many patients have absolute or relative contraindications to DOAC. The LILAC -TIMI 76 trial (ClinicalTrials.gov ID No. NCT05712200**)** is an ongoing randomized, double blind phase 3 trial investigating the efficacy and safety of the FXI inhibitor abelacimab, given subcutaneously once a month, compared to placebo in ~ 1,900 patients with AF deemed unsuitable for anticoagulation, which is still recruiting. After the publication of these phase 3 trials, further publication of subgroup analyses, including by age, risk factors for bleeding, degree of renal function and concomitant antiplatelet therapy will help identify the optimal niche for FXI/XIa inhibitors in treating patients with AF.

### Limitations

The strength of our study is the consistent reporting of the primary outcome of major bleeding endpoints across the three trials included and the low risk of bias for all studies, suggests that the findings are unlikely to be affected by systematic errors or study design flaws. However, our results should be considered in relation to both general limitations of meta-analyses, as well as the specific limitations of this study. Due to the small number of studies available, together with heterogeneity in the pharmacokinetics of the drugs and comparators, our analysis should be interpreted with caution. Importantly, two of the 3 studies included were not individually powered to assess the effects on ischemic endpoints, so our findings in that regard have to be considered exploratory. While that is a limitation, this is also where a meta-analysis may be helpful. Follow up duration varied in the studies, and notably, OCEANIC-AF was terminated prematurely on the advice of the Independent Data Monitoring Committee due to an inferior efficacy of asundexian cmpared with apixaban for the prevention of stroke and systemic embolism.

The studies included two drugs with different mechanisms of action, namely FXI vs. FXIa inhibition, different routes of administration (oral versus subcutaneous), and very different half-lives, as well as different dosages. Furthermore, the comparator DOAC was asundexian in the PACIFIC-AF and OCEANIC-AF trials [17, 18], a drug given twice daily with a half-life of approximately 13 hours [29] and rivaroxaban, given once daily with a half-life of 5–13 hours [29] in the AZALEA-TIMI 71 study [19]. 

The primary endpoint in all trials included ISTH major bleeding, but only the two phase 2 trials also evaluated CRNM bleeding. We used trial-level data for outcomes assessment; hence, we could not conduct an in-depth analysis of major bleeding events (e.g. gastrointestinal bleeding, fatal bleeding). We have not analyzed some patient-level characteristics (e.g. specific bands of CHA_2_DS_2_VASc score, hypertension, advanced age) or specific clinical setting (e.g. post-PCI, patients with cancer, prior bleeding) where the benefit of reduced bleeding risk may be particularly important. Specifically, in trial level data available, there is insufficient information provided on subgroups such as the CHA_2_DS_2_VASc score, bleeding risk score, breakdown by renal function or age bands of enrolled patients to be able to perform such subgroup analyses.

## Conclusion

Compared to treatment with a DOAC, FXI inhibition significantly reduced the incidence of ISTH major bleeding, CRNM bleeding or the composite of these. Exploratory analyses indicate similar risk of intracranial hemorrhage, but possible increased ischemic stroke risk with FXI inhibitors compared to DOAC. The results of ongoing trials are awaited to build on this early evaluation of the safety and efficacy of FXI inhibitor use in patients with AF.

## Electronic supplementary material

Below is the link to the electronic supplementary material.

Supplementary Material 1

## Data Availability

No datasets were generated or analysed during the current study.

## Abbreviations

AF: Atrial fibrillation
CRNM: Clinically relevant non-major
DOAC: Direct oral anticoagulant
TF: Tissue factor
VTE: Venous thromboembolism
