# Supplementary material for: Safety of factor XI inhibitors compared to factor X inhibitors in atrial fibrillation: a systematic review and meta-analysis
Source: J Thromb Thrombolysis. 2025 Jul 3;58(7):761–73. doi: 10.1007/s11239-025-03142-x (PMC12612016; doi:10.1007/s11239-025-03142-x)
Supplement: Supplementary file 1 — Supplementary Material 1 [file 11239_2025_3142_MOESM1_ESM.docx]

**Response to the Reviewer(s)**

We thank the reviewer(s) for taking the time to review our manuscript and for the helpful comments and recommendations. Please find below a point-by-point response to the reviewer’s comments (in blue).

1. Expand on Clinical Implications • Clarify the clinical relevance of the findings, especially how FXI inhibitors might be used in practice. • Discuss which patient populations (e.g., high bleeding risk, elderly, post-PCI) might benefit most from FXI inhibitors based on the available data and knowing the potential bleeding risk for patients with AF.

We thank the reviewer for this very helpful suggestion. We have therefore inserted the following paragraph on p9:

“The relevance of these findings is that there is potentially a new anticoagulant strategy to reduce major bleeding compared to standard-of-care (FXa inhibitors), and whilst our study specifically analyzed patients with an indication for anticoagulation for thromboprophylaxis of AF, other patients with indications for anticoagulation may also benefit from such a strategy to reduce major bleeding. This includes patients with as venous thromboembolism, particularly patients with cancer, as well as for secondary prevention of stroke and in patients with acute coronary syndrome.^27^ Such a strategy to reduce bleeding may be particularly relevant for patients with AF who have had a prior bleed on a DOAC, or are at increased risk of bleeding.^28^  The risk of OAC-related bleeding increases with age and includes those with uncontrolled hypertension, solid tumors or hematological malignancies, liver or renal dysfunction, prior stroke or cerebral small vessel disease or amyloid angiopathy, those with anemia, thrombocytopenia, those the excessive alcohol intake or those who require concomitant antiplatelet medications. Additionally, there is undeniably a cohort who are undertreated with DOAC because of concerns around frailty and falls-risk, in whom the lower risk of bleeding afforded by FXIa inhibitor compared to that with DOACs, may favor the use of thromboprophylaxis. The exploratory analyses indicating similar risk of intracranial hemorrhage with FXI/FXIa inhibitors and DOACs, if borne out in the ongoing Phase 3 studies, would imply that use of FXIa inhibitors would not be preferred over DOACs in those with prior stroke, cerebral small vessel disease or amyloid angiopathy. Furthermore, DOACs would continue to be preferrable over FXI/FXIa inhibitors for those at high ischemic risk, and/or those at low risk of bleeding risk, but FXIa inhibitors may be preferred for those at lower ischemic and higher bleeding risks.”

2. Address Mechanistic Insights • Provide more discussion on the biological rationale for the observed increased ischemic stroke risk with FXI inhibitors. Does this speak to the target, drug(s) or both?

We are grateful to the reviewer for this very helpful suggestion. We have accordingly inserted the paragraph below on p8:

“The reason for the increased risk of ischemic stroke seen with FXIa inhibition compared to DOAC is unclear, but some possible mechanisms are worthy of discussion. Firstly, in dose-finding studies, the effectiveness of asundexian was gauged by the degree of FXI inhibition. However, the extent of FXI inhibition may not directly reflect efficacy of the drug on thrombin generation or even on fibrin generation. A differential impact of apixaban and asundexian on thrombin generation is supported by pharmacodynamic studies in vitro.^20^ Furthermore, pharmacokinetic studies showed that the inhibition constant (Ki) of asundexian for FXIa is 1.0 nM,^20^ whereas the Ki for apixaban against FXa is 0.08 nM.^21,22^ This may be pertinent for dosing. While the half-lives of milvexian and asundexian are roughly similar, the OCEANIC-AF trial with asundexian was terminated prematurely for non-inferiority while the phase 3 trial with milvexian, LIBREXIA-AF has finished recruiting and is now in follow-up(x), without premature closure due to efficacy concerns. However, it is noteworthy that the selected dose of milvexian in LIBREXIA-AF (ClinicalTrials.gov ID No. NCT05757869) at 100 mg twice daily is 4-fold higher than the dose of asundexian studied in OCEANIC-AF (50 mg daily). Furthermore, milvexian has been reported to have greater potency in the FXIa enzyme inhibition assay in vitro compared to that reported for asundexian.^23,24^ Lastly, perhaps the setting of AF may not be the right thrombotic target for FXI/FXIa inhibition. Since FXI inhibitors target the intrinsic pathway of coagulation, it is not surprising that early studies in vitro showed that asundexian was less effective at inhibiting thrombin generation in the presence of high concentrations of tissue factor (TF) compared to that when low amounts of TF were used as trigger.^24^ It is possible that in patients with AF, the contribution of the extrinsic (TF) pathway of coagulation has been underestimated and TF may play a more significant role than previously thought.”

Include hypotheses or mechanistic models that could explain the mortality benefit despite higher stroke risk.

We thank the reviewer for this very helpful recommendation. Accordingly, we have expanded the following paragraph on p9:

“All-cause mortality was lower with FXI inhibition than DOAC. The mechanism behind this is unclear, although likely to at least in part relate to the lower risk of major bleeding with FXI inhibition. Additionally, the lower rate of minor/nuisance bleeding reported with FXI inhibition could have resulted in better treatment compliance in patients assigned to FXI inhibitor than those taking DOAC, leading to lower risk of venous thromboembolism.

Finally, possible off-target beneficial effects of FXI inhibition have to be considered. FXI activation promotes a proinflammatory phenotype and FXI inhibition in non-human primates was shown to reduce C-reactive protein, platelet reactivity and endothelial cell activation.^25^  Additionally, in vitro studies have shown that FXI activation increases vascular permeability, a recognized feature of inflammation, and inhibition of FXI activation was shown to preserve endothelial barrier function.^26”^

Deepen Subgroup Analyses • If possible, with published rather than patient-level data, explore more granular subgroup analyses, such as stratification by CHA₂DS₂-VASc or HAS-BLED scores.

We have accordingly expanded the “Limitations” section to now include:

“We have not analyzed some patient-level characteristics (e.g. specific bands of CHA_2_DS_2_VASc score, hypertension, advanced age) or specific clinical setting (e.g. post-PCI, patients with cancer, prior bleeding) where the benefit of reduced bleeding risk may be particularly important. Specifically, in trial level data available, there is insufficient information provided on subgroups such as the CHA_2_DS_2_VASc score, bleeding risk score, breakdown by renal function or age bands of enrolled patients to be able to perform such subgroup analyses.”

Clarify Limitations and Future Directions • Be more explicit about how ongoing trials (e.g., LIBREXIA-AF) might address current uncertainties. • Suggest specific research questions for future studies, such as optimal dosing or combination with antiplatelets.

We thank the reviewer for these helpful suggestions.

We have accordingly expanded the “Limitations” section on p11 to now include:

“We have not analyzed some patient-level characteristics (e.g. specific bands of CHA_2_DS_2_VASc score, hypertension, advanced age) or specific clinical setting (e.g. post-PCI, patients with cancer, prior bleeding) where the benefit of reduced bleeding risk may be particularly important. Specifically, in trial level data available, there is insufficient information provided on subgroups such as the CHA_2_DS_2_VASc score, bleeding risk score, breakdown by renal function or age bands of enrolled patients to be able to perform such subgroup analyses.”

We have expanded the “Discussion” section to now include:

“An ongoing large, double-blind, randomized Phase 3 trial (LIBREXIA-AF) is evaluating another small molecule oral FXIa inhibitor, milvexian, compared to asundexian, to reduce the risk of the composite stroke and non-central nervous system systemic embolism in ~20,000 patients with AF (ClinicalTrials.gov ID No. NCT05757869). The trial has finished recruiting and is in follow up. Importantly, compared to OCEANIC-AF, where asundexian was given as a once-a-day 50mg dose, in LIBREXIA-AF, milvexian is given at a dose of 100mg twice daily, in effect fourfold higher dose per 24 hour period than for asundexian in OCEANIC-AF. This will help clarify the effectiveness and safety of higher dose oral FXIa inhibition compared to DOAC. Furthermore, many patients have absolute or relative contraindications to DOAC. The LILAC -TIMI 76 trial (ClinicalTrials.gov ID No. NCT05712200**)** is an ongoing randomized, double blind phase 3 trial investigating the efficacy and safety of the FXI inhibitor abelacimab, given subcutaneously once a month, compared to placebo in ~1,900 patients with AF deemed unsuitable for anticoagulation, which is still recruiting. After the publication of these phase 3 trials, further publication of subgroup analyses, including by age, risk factors for bleeding, degree of renal function and concomitant antiplatelet therapy will help identify the optimal niche for FXI/XIa inhibitors in treating patients with AF.”
